# FDA validated ecofriendly HPTLC method for quantification of Florfenicol and Meloxicam in bovine tissues with sustainability assessment

**DOI:** 10.1038/s41598-025-18548-z

**Published:** 2025-09-15

**Authors:** Marco M. Z. Sharkawi, Eglal A. Abdelaleem, Manar A. Mohamed, Mohamed Ahmed Elsayed

**Affiliations:** 1 Pharmaceutical Analytical Chemistry Department, Faculty of Pharmacy, Beni-Suef University, Beni-Suef, Egypt; 2 Pharmaceutical Analytical Chemistry Department, Faculty of Pharmacy, Fayoum University, Fayoum, Egypt

**Keywords:** Florfenicol, Meloxicam, HPTLC, Bioanalysis, Eco-friendly assessment, Biochemistry, Biological techniques, Chemistry, Drug discovery

## Abstract

Meloxicam and Florfenicol, two pharmacologically distinct agents with anti-inflammatory and antibacterial properties, respectively, have attracted considerable interest in veterinary medicine due to their therapeutic efficacy and safety profiles, particularly in cattle and poultry. In this study, a cost-effective and straightforward thin-layer chromatography (HPTLC) densitometric method was developed for the simultaneous Quantification of these drugs in both pharmaceutical formulations and spiked bovine muscle samples, with the aim of monitoring tissue residues. The chromatographic separation was achieved using a mobile phase consisting of glacial acetic acid, methanol, triethylamine, and ethyl acetate (0.05: 1.00: 0.10: 9.00, by volume). Densitometric detection was performed at 230 nm, with Esomeprazole (ESO) employed as an internal standard to compensate for potential wavelength fluctuations. Method validation was conducted in accordance with ICH guidelines, demonstrating Linearity within the ranges of 0.03–3.00 µg/band for meloxicam and 0.50–9.00 µg/band for Florfenicol. This method offers a reliable analytical tool for regulatory and surveillance purposes, contributing to public health protection and food safety by enabling the detection of veterinary drug residues in edible tissues. The environmental impact of the method was evaluated using five greenness assessment tools, including greenness, whiteness, and blueness metrics, confirming its eco-friendly nature.

## Introduction

 Veterinary pharmaceuticals can be broadly categorized into therapeutic agents (e.g., antibiotics and anthelmintics) and nutritional or growth-promoting supplements (e.g., hormones). These drugs are integral to animal health management, disease control, and productivity enhancement, and are routinely used in both clinical and agricultural settings. However, the improper or excessive use of such substances can result in the accumulation of drug residues in animal-derived food products, such as meat, milk, and eggs, posing potential health risks to consumers. The presence of residual veterinary drugs in animal products remains a critical public health concern^1^. Antibiotics are among the most commonly used veterinary drugs due to their ability to inhibit or eliminate pathogenic microorganisms, even at low concentrations. Common classes of veterinary antibiotics include β-lactams, tetracyclines, penicillin, streptomycin, and tylosin^2^. These compounds can leave behind residues—either the parent compound or its metabolites—in edible animal tissues^3^. If not adequately regulated, these residues can accumulate and biomagnify through the food chain, potentially posing health risks to humans. The US Food and Drug Administration (FDA) defines drug residues as any compound found in edible tissues resulting from the administration of a drug, including the drug itself, its metabolites, and related substances. Under Sect. 556 of Title 21, the FDA sets tolerance levels for residues of approved veterinary drugs^4^. Similarly, the European Union regulates maximum residue limits (MRLs) for pharmacologically active substances in animal-derived food products under Commission Regulation (EU) No. 37/2010^5^. This study investigates the combined effects of Florfenicol (FLR) and meloxicam (MEL), administered in the prescription formulation Zeleris^®^, on muscle development in cattle. Florfenicol, a broad-spectrum antibiotic used in both terrestrial and aquatic veterinary medicine, acts by inhibiting bacterial protein synthesis via ribosomal inactivation. It also exhibits anti-inflammatory properties by suppressing immune cell proliferation and cytokine release^6^. Meloxicam, a nonsteroidal anti-inflammatory drug (NSAID) of the enolic acid class, primarily inhibits cyclooxygenase-2 (COX-2) and is used to manage inflammatory conditions such as osteoarthritis and rheumatoid arthritis^7^. The European Commission has set MRLs of 200 µg/kg for FLR and 20 µg/kg for MEL in bovine muscle tissue^8^. These values are consistent with Codex Alimentarius guidelines, while the US FDA permits up to 300 µg/kg for FLR and has not established an MRL for MEL. In Egypt, official MRLs are not publicly available; therefore, EU or Codex standards are commonly referenced for regulatory compliance and food safety assessments. Increased consumption of veterinary drugs such as meloxicam (MEL) and Florfenicol (FLR) raises concerns regarding allergic reactions and the development of antimicrobial resistance in humans. Samples of bovine muscle tissue were procured from a local purveyor in Fayoum, Egypt. The purveyor guaranteed that the cattle from which the samples were taken had not been administered any pharmaceutical agents prior to slaughter, which was consistent with established regional protocols. Several analytical methods have been reported for the individual determination of MEL and FLR in various matrices. Thin-layer chromatography (TLC) has been applied for MEL analysis^9–12^, along with spectrophotometric, fluorimetric, and high-performance liquid chromatography (HPLC) techniques^7^, and a high-performance thin-layer chromatographic method^13^. FLR has been quantified using spectrophotometric methods^14^, HPLC^15–18^, and liquid chromatography–tandem mass spectrometry (LC-MS/MS)^19^. However, these methods typically target a single analyte and are not validated for simultaneous detection in complex matrices like bovine tissue. To address this gap, we developed and validated a cost-effective HPTLC-densitometric method for the simultaneous Quantification of MEL and FLR in spiked cattle muscle. Table 1 summarizes key differences between published methods and the proposed approach.

**Table 1:** Comparison of analytical techniques for the detection and quantification of meloxicam (MEL) and florfenicol (FLR) in various matrices using different detection methods.

Ref. No.	Analyte	Matrix	Technique	LOD	LOQ
proposed HPTLC	MEL & FLR	Cattle muscle	HPTLC-Densitometry	0.06 µg/spot (MEL), 0.18 µg/spot (FLR)	0.17 µg/spot (MEL), 0.56 µg/spot (FLR)
^10^ Parys et al., 2021	MEL	Tablets	TLC-Densitometry	0.25 µg/spot	~ 0.75 µg/spot*
^12^ Hopkała & Pomykalski, 2003	MEL	Tablets	TLC-densitometry	~ 0.20 µg/spot	~ 0.60 µg/spot*
^7^ Hassan, 2002	MEL	Formulations	Spectrophotometry/Fluorimetry	~ 0.10 µg/mL	~ 0.30 µg/mL*
^13^ Bae, J.-W,2017	MEL	Bulk drug & dosage form	HPTLC-Densitometry	0.03 µg/spot	0.099 µg/spot
^14^ Elimam et al., 2016	FLR	Bulk drug	Spectrophotometry	0.25 µg/mL	~ 0.75 µg/mL*
^15^ Zhou et al., 2020	FLR	Porcine tissues	TLC-HPLC	0.05 µg/g	0.15 µg/g
^16^ Hayes, 2005	FLR	Fish feed	HPLC	0.03 µg/g	0.10 µg/g
^17^ Nasim et al., 2016	FLR	Poultry meat & liver	RP-HPLC	0.01–0.05 µg/g	0.05–0.15 µg/g
^18^ Granja et al., 2012	FLR	Fish muscle	HPLC-UV & LC-MS/MS	0.02 µg/g	0.06 µg/g

## Materials and methods

### Chemicals and reagents

Meloxicam and Florfenicol are supplied by Delta Pharma Association for Medications and Chemicals in Cairo, Egypt. For MEL and FLR, their purity was 99.95% and 98%, respectively, based on an analysis from the manufacturer’s certificate. Esomeprazole (ESO), the internal standard (IS), was provided by the Chemi Pharm Association for Medications and Chemicals in Cairo, Egypt. Its purity was verified to be 100.05%, and it was examined using the approved procedures. Triethylamine and HPLC-grade methanol were purchased from Fischer, London, UK. Glacial acetic acid and ethyl acetate were acquired from EL-Nasr Pharmaceutical, Chemical Co. (Cairo, Egypt). Sodium hydroxide and EDTA were purchased from Sigma-Aldrich (Germany) and were of analytical grade. Zeleris^®^ is an injectable solution that contains: 5 mg/mL of meloxicam and 400 mg/mL of Florfenicol. The product was sourced from the local market and manufactured by Ceva Sante Animale. All sample solutions were filtered using a 0.45 μm nylon membrane filter (25 mm diameter) purchased from Sigma Aldrich.

### Apparatus

Aluminum 20*20 cm HPTLC plates covered with 5 μm particle size silica gel 60 F254 and a thickness of 0.25 mm (Merck, Germany) were utilized. Applications were carried out with a Camag Linomat IV applicator and a 100 mL syringe. The HPTLC scanner used for the scanning was a model 3 S/N Camag (Muttenz, Switzerland) linked to and operated by WinCATS software (version 3.15).

### HPTLC densitometric conditions

Using a CAMAG Linomat V applicator, internal standard (ESO) and sample medications were applied on HPTLC plates to perform the HPTLC-densitometric technique. In a dual-trough chamber that had been earlier saturated for 15 min with a developing system that consisted of glacial acetic acid, methanol, tri-ethyl amine, and ethyl acetate (0.05: 1.00: 0.10: 9.00, by volume) at room temperature employing a UV lamp with a wavelength of 230 nm.

### Preparation of solutions

#### Stock solutions

Florfenicol (FLR) at a concentration of 5000 µg/mL, Meloxicam (MEL) at 1000 µg/mL, and Esomeprazole (ESO) at 1000 µg/mL. Each solution was dissolved in 0.50 mL 1 N NaOH and completed with methanol in distinct 25 mL calibration flasks.

#### Working solutions

Florfenicol (FLR) at a concentration of 1000 µg/mL and meloxicam (MEL) at a concentration of 100 µg/mL were diluted from their earlier cited stock solutions distinctly in 10 mL volumetric flasks using methanol.

#### Pharmaceutical formulation

A stock solution of $\:{Zeleris\:}^{\circledR\:}$(a solution for injection) was prepared by precisely taking.125mL from the drug and transferring it into a 25 mL glass volumetric flask. 0.50 mL 1 N NaOH was added, and methanol was added to bring the volume up to the mark, resulting in stock solutions with concentrations of 2000 µg/mL (FLR) and 25 µg/mL (MEL). From this primary stock solution, 2 mL solution was transferred into 10 mL volumetric flasks. 0.50 mL of ESO from its stock solution was added, followed by methanol to achieve the final dilution.

### Linearity and calibration curves

#### Pure samples

Calibration curves were produced After serially diluting FLR and MEL in the ranges of 0.50–9.00 µg/band and 0.03–3.00 µg/band, respectively, from stock solutions (5000 µg/mL for FLR and 1000 µg/mL for MEL), in two separate groups of 10 mL volumetric flasks then 0.50 mL of ESO at the concentration (1000 µg/mL) was added to each sample subsequently ten microliters of each sample were spotted in triplicate onto the HPTLC plates after the volume was adjusted to ten milliliters using methanol and filtration the sample. Next, chromatographic separation was performed as previously described for each component. Integrated Peak area (the peak area of the analyte/peak area of IS) was measured and utilized to analyze the data. Regression equations were then calculated by creating calibration curves that connected the calculated integrated peak area to a suitable concentration.

#### Spiked muscle sample

The same concentration levels that were previously discussed under calibrations for pure samples FLR and MEL in the ranges of 0.50–9.00 µg/band and 0.03–3.00 µg/band, respectively, were used to generate the calibration standards. The method was validated by analyzing spiked quality control (QC) samples of (FLR) and (MEL). These samples were prepared at low, medium, and high concentrations to ensure comprehensive validation. Specifically, (MEL) was present at 0.30, 1.50, and 2.40 µg/band, while (FLR) was at 2.00, 4.00, and 7.00 µg/band. All these concentrations fell within the established calibration range. Two grams of cattle muscle were obtained from a local butcher in Fayoum, Egypt. The seller assured that the animals were not administered any pharmaceutical agents prior to slaughter, which was consistent with standard regional practices. The muscle was thoroughly homogenized in mortars for each sample preparation. After that, they were moved into several tubes and spiked individually with the prescribed quantity of each medication. Each sample was treated with 300 µl of 0.10 N EDTA, 0.50 mL of ESO (1000 µg/mL), and methanol to complete the volume (5.00 mL). The samples were thoroughly vortexed for 4 min using a 250 VM vortex mixer (Hwashin, Seoul, Korea). To remove the precipitated proteins and fats, they were centrifuged at 1789 ×g for 15 min at 2℃ (Zjmzym, China), then the clear supernatant was moved to a new batch of test tubes with a capacity of ten milliliters. After applying 5 mL of methanol to the preserved tissue and agitating it violently for more than 4 min, the tissue was centrifuged for 15 min at 1789 ×g. For the whole removal of the medications. The supernatant was then pooled with the first one as a supplement. Next, methanol was added until the level reached 10 mL. Samples were subsequently added to HPTLC plates (10 µl) after filtration, and adherence to the chromatographic conditions was ensured. Calibration curves were formed after the recording of peak area ratios.

#### Quality control samples (QCs)

0.30, 1.50, and 2.40 µg/band of MEL, and 2.00, 4.00, and 7.00 µg/band of FLR were the quality control samples (QCs) that were made similarly to calibration-spiked tissue samples and subsequently utilized under FDA criteria to assess the new method’s validity.

#### Pharmaceutical formulation

For application, dosage form concentrations were used: for FLR, and MEL 4.00: 0.50 µg/band. Then 0.50 mL (1000 µg/mL) IS was added to it. Each was administered in triplicate on HPTLC plates in an amount of 10 µL. Each drug’s peak area ratio was calculated, and the matching concentrations in the formulated pharmaceutical dosage form solutions were determined using the regression equation. Also, for every medication, there were three levels of use of the standard addition technique.

### Method validation parameters

The analytical method was validated in accordance with the ICH M10 guideline on Bioanalytical Method Validation. The validation parameters assessed included selectivity, linearity, accuracy, precision, recovery, matrix effect, and stability. Linearity was evaluated using calibration curves with a minimum of six concentration levels, requiring a correlation coefficient (r2) of ≥ 0.99. Accuracy and precision were assessed at multiple quality control (QC) levels. The acceptance criteria were ± 15% deviation from nominal concentrations for accuracy and ≤ 15% relative standard deviation (%RSD) for precision. However, at the lower Limit of Quantification (LLOQ), a deviation of ± 20% was acceptable. Recovery and matrix effects were evaluated to ensure consistent analyte extraction and to confirm that there was no interference from components of the biological matrix. Stability was confirmed through studies conducted under various conditions, including bench-top, freeze-thaw, and long-term storage, to ensure sample integrity.

## Results and discussion

### Method optimization

#### Sample extraction

Beef is a high-quality protein source containing all essential amino acids. It is also rich in bioavailable iron, zinc, and vitamin B12—nutrients vital for immune function, oxygen transport, and neurological health. Compared to plant-based sources, beef offers superior nutrient bioavailability, making it particularly valuable in populations at risk of deficiency^20^.

Cattle muscle tissue is primarily composed of water (75%), proteins (20%), lipids (5%), minerals (5%), and vitamins (5%)^21^. During extraction, ethylenediaminetetraacetic acid (EDTA) was used as a potent chelating agent to sequester metal ions bound to muscle metalloproteins^22^. Based on previously reported protocols^23–25^, 300 µL of 0.10 N EDTA was added for effective metal chelation. For protein and lipid precipitation, organic solvents such as methanol and acetonitrile were evaluated^26^. Among these, methanol alone produced the clearest densitograms and was therefore selected as the optimal solvent. The extraction was optimized through a two-step methanol-based procedure to enhance analyte recovery. Initially, 5 mL of methanol was added to the homogenized sample, followed by centrifugation at 1789 ×g for 15 min. The resulting pellet was then treated with an additional 5 mL of methanol and subjected to a second centrifugation under the same conditions. The supernatants from both steps were pooled and analyzed using the proposed high-performance thin-layer chromatography (HPTLC) densitometric method. This extraction protocol was characterized by high recovery efficiency and minimal solvent consumption.

#### Mobile system

The HPTLC separation was performed using silica gel 60 F₂₅₄ plates. To identify an effective mobile phase, various solvent systems were initially evaluated, including toluene: methanol, methanol: methylene chloride, methanol: chloroform, and methanol: ethyl acetate (all in 5:5 v/v ratios). Among these, methanol: ethyl acetate showed the most promising results. Further optimization was carried out by varying the ratio of ethyl acetate to methanol from 9:1 to 5:5 (v/v). The optimal resolution and retention factor (Rf) values for both meloxicam (MEL) and Florfenicol (FLR) were achieved with a mobile phase consisting of ethyl acetate: methanol (9:1, v/v). However, MEL exhibited tailing with asymmetric peak shapes under these conditions. To improve peak symmetry, the mobile phase was modified by adjusting pH. Various concentrations of ammonium hydroxide (0.10–0.50) ml were tested, but the tailing issue was not resolved. Subsequently, glacial acetic acid and triethylamine were added to the mobile phase. Triethylamine reduces tailing of basic compounds by neutralizing acidic silanol groups on the stationary phase, thereby enhancing spot clarity and resolution^27^. Glacial acetic acid protonates basic sites, further reducing interactions with the silica surface and improving separation efficiency for basic analytes^28^.

#### Internal standard

To correct for variability in analyte recovery and potential losses during sample preparation and analysis, the use of an internal standard (IS) is essential in the development of bioanalytical methods. Several candidate compounds were evaluated as potential internal standards, including celecoxib, metformin, ivermectin, and oxytetracycline. Among these, esomeprazole demonstrated the most favorable chromatographic behavior and resolution under the optimized HPTLC conditions, making it the most suitable choice as the internal standard for this study.

#### The scanning wavelength

Various scanning wavelengths (230, 254, 270, and 360 nm) were evaluated to determine the optimal detection sensitivity for meloxicam (MEL) and Florfenicol (FLR). Among these, 230 nm provided the highest overall sensitivity for both analytes. At 254 and 270 nm, the sensitivity for FLR was significantly reduced, while at 360 nm, only MEL was detectable, with no visible response for FLR.

### Method development

As per the instructions mentioned in the “chromatographic conditions” section, the calibration curves were made when plotting the peak area ratio, which is calculated by dividing the peak area of the drug by the peak area of IS, against the appropriate concentration in µg/band. In the MEL and FLR concentration ranges of 0.03–3 µg/band and 0.50–9 µg/band, respectively, linear regressions were observed. The regression equations’ standards are listed in Table 2.

**Table 2:** Assay and method validation parameters for the determination of meloxicam (MEL) and florfenicol (FLR) by the proposed HPTLC method.

Parameters	Pure		Spiked muscle	
	MEL	FLR	MEL	FLR
Calibration range (µg/band)	0.03–3.00	0.50-9.00	0.03-3.00	0.50-9.00
Slope	94.04	31.03	94.17	31.10
Intercept	91.98	109.29	91.47	108.51
Correlation coefficient	0.9999	0.9998	0.9999	0.9998
Accuracy (mean)	98.90	100.84	99.78	100.97
Robustness parameters (RSD% %)^a^	-			
-tri ethyl acetate amine(0.1 ± 1% mL)			1.54	1.71
-detection wavelength (230 ± 0.5 nm)			1.62	1.67
- saturation time (15 ± 5 min)			1.59	1.55
LLOQ (µg/band) ULOQ (µg/band)			0.03 3.00	0.50 9.00

The (RS$\:{D\%)}^{a}$was calculated for R_f_ values, and it is the average of three determinations.

Notes: LLOQ, the lower Limit of Quantification; RSD, relative standard deviation; SD, standard deviation; ULOQ, upper Limit of Quantification.

### Method validation

The established bioanalytical HPTLC technique was proven by adhering to the [ICH M10 guidelines.

#### Calibration curves, lower and upper limits of quantification

For MEL and FLR, concentration ranges (0.03–3.00 µg/band and 0.50–9 µg/band) were defined for linear calibration curves of spiked muscle samples with the medicines under study.

The LLOQ and the ULOQ quantitation Limits were discovered to be 0.03 and 3.00 µg/band for MEL and 0.50 and 9.00 µg/band for FLR, as presented in Table 2.

#### Accuracy, precision, and quality control samples

LLOQ, LQC, MQC, and HQC were the four QC samples that were validated to evaluate the precision and accuracy of the established procedure. The QC concentrations that were chosen were 0.03, 0.30, 1.50, and 2.40 µg/band, regarding MEL, 0.50, 2.00, 4.00, and 7.00 µg/band concerning FLR. The matching regression equations, as provided in Table 2, were employed to determine the concentrations of the medicines under study. The results obtained were deemed satisfactory, as presented in Table 3.

**Table 3:** Intra- and inter-day precision and accuracy of LLOQ, LQC, MQC, and HQC of meloxicam and florfenicol in spiked muscle samples.

Concentration(µg/band)^b^		Intra day			Inter day		
		Recovery %	RSD %	Bias %^a^	Recovery %	RSD %	Bias %^a^
MEL	0.03 LLOQ	100.26	1.24	0.26	99.72	1.26	0.20
	0.30 LQC	100.14	0.17	0.18	101.50	0.25	0.30
	1.50MQC	98.28	0.07	–1.66	96.44	0.08	–2.67
	2.40HQC	100.73	0.06	0.72	98.32	0.11	0.76
FLR	0.50LLOQ	102.60	0.27	2.41	104.03	0.29	2.03
	2.00LQC	101.22	0.30	1.05	96.79	0.34	0.09
	4.00MQC	99.04	0.05	–0.95	103.48	0.10	–0.18
	7.00HQC	98.85	0.07	−1.15	98.46	0.09	−0.99

(a) Bias%= [(measured concentration - nominal concentration)/nominal concentration] * 100.

(b) Average of five experiments.

Notes: HQC, the high-quality control; LLOQ, the lower Limit of Quantification; LQC, low-quality control; MQC, the middle-quality control; RSD, relative standard deviation.

#### Specificity and selectivity

**Fig. 1:**
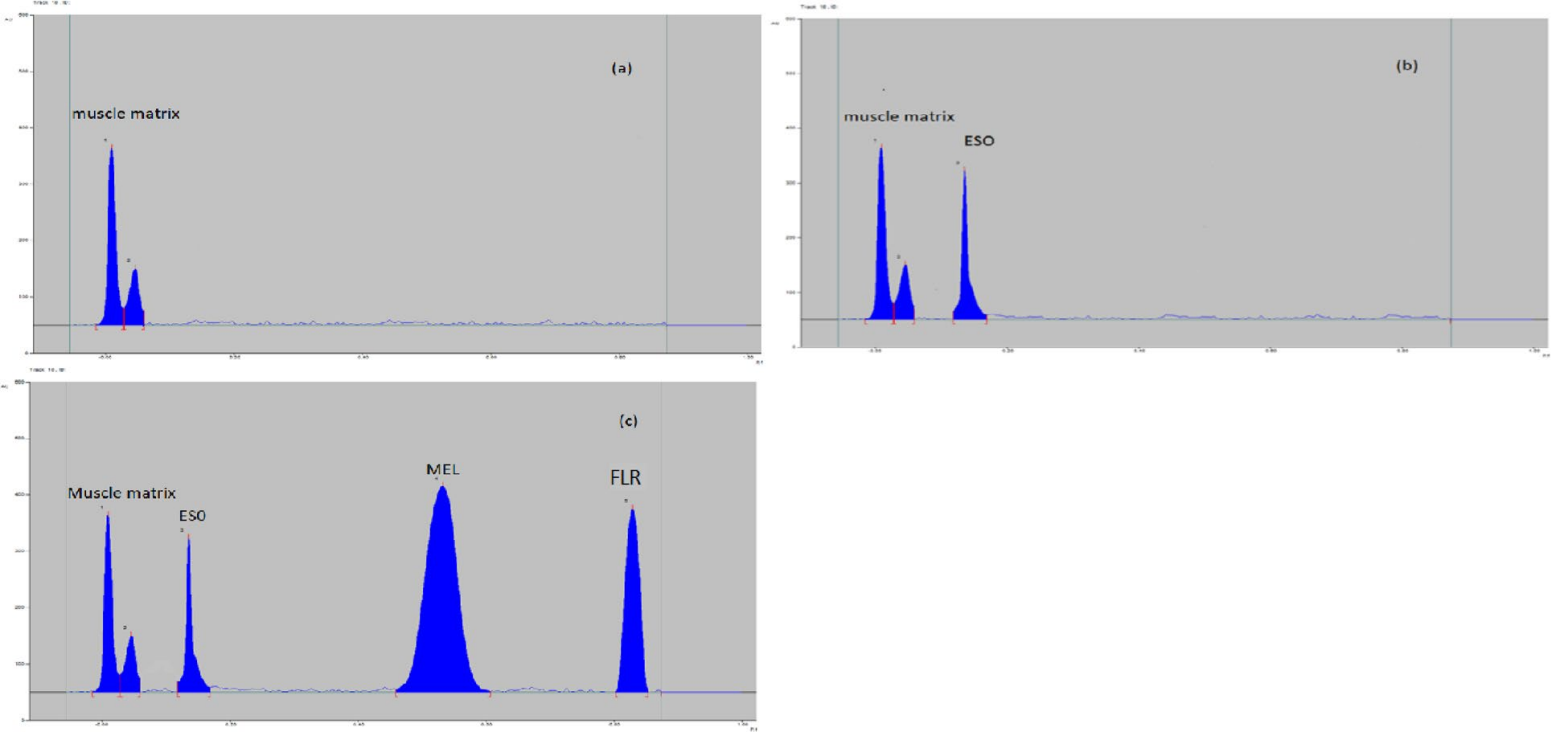
2D chromatogram of **(a)** blank muscle sample; **(b)** blank muscle sample and 0.5 µg/band of the internal standard (IS) esomeprazole;**(c)** spiked muscle sample with 7 µg/band of florfenicol, 2.4 µg/band of meloxicam, and 0.5 µg/band of the IS esomeprazole, using glacial acetic acid, methanol, tri-ethyl amine, and ethyl acetate, (0.05: 1.00: 0.10: 9.00, by volume) as a developing system at 230 nm.

**Fig. 2:**
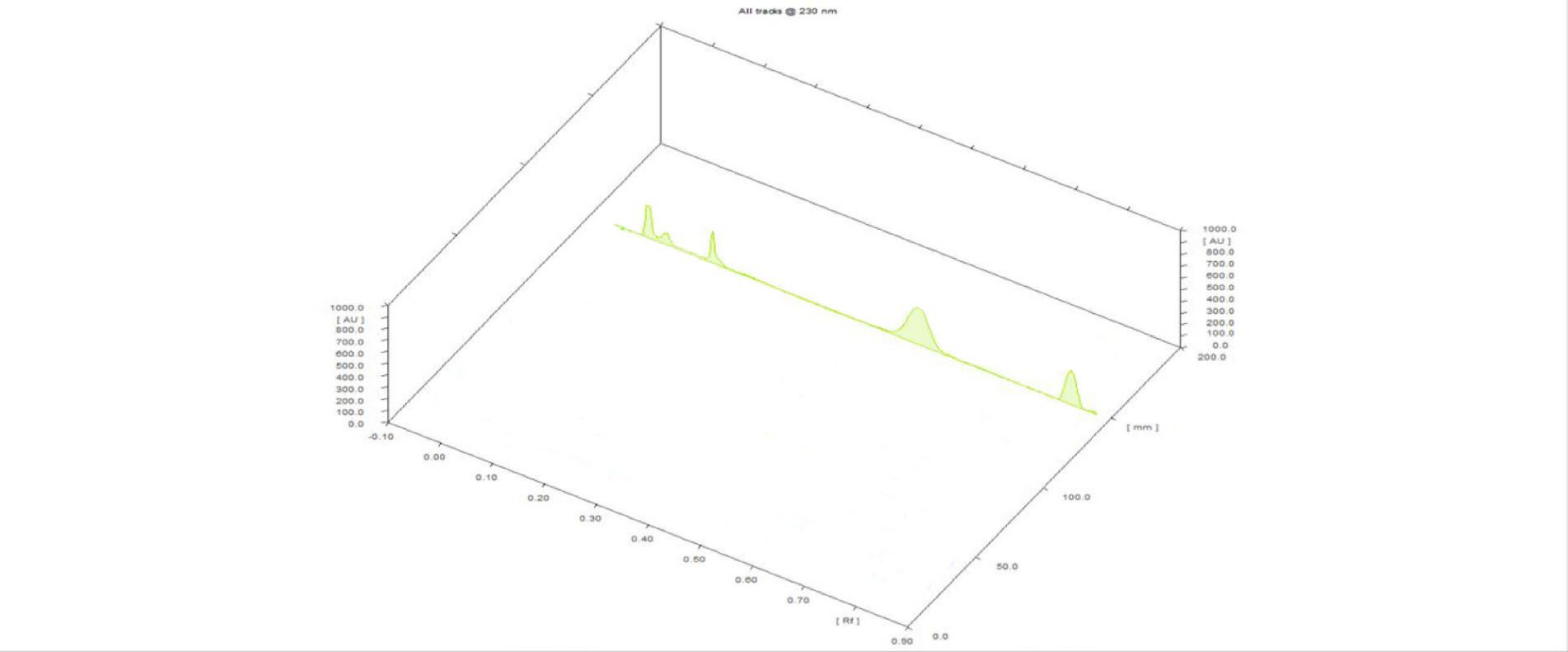
3D chromatogram of **(a)** blank muscle sample; **(b)** blank muscle sample and 0.5 µg/band of the internal standard (IS) esomeprazole; **(c)** spiked muscle sample with 7 µg/band of florfenicol, 2.4 µg/band of meloxicam, and 0.5 µg/band of the IS esomeprazole, using glacial acetic acid, methanol, tri-ethyl amine, and ethyl acetate, (0.05: 1.00: 0.10: 9.00, by volume) as a developing system at 230 nm.

To verify the specificity, HPTLC chromatograms obtained from the application of blank muscle samples and spiked muscle samples containing the two medicines under study at their HOQ and IS were compared. As evidence of the new method’s selectivity, (Figs. 1 and 2) HPTLC chromatograms demonstrated good separation between the muscle samples, MEL, FLR, and IS. No significant interfering peaks were observed at the respective Retention factor (Rf) values. To further evaluate the method’s accuracy and assess the matrix effect, a standard addition recovery study was performed. Blank muscle samples were spiked with known quantities of (MEL) and (FLR) at three concentration levels: low, medium, and high, spanning the calibration range. Each spiked sample was subjected to the same extraction protocol detailed in Sect. “Spiked muscle sample”. The calculated recoveries, expressed as the percentage of the added analyte recovered, were 104.58% for MEL and 98.97% for FLR, as shown in Table 4. These results confirm the method’s accuracy even in the presence of matrix components and indicate minimal interference from excipients, particularly when applied to the commercial formulation Zeleris^®^ (solution for injection). These findings are further corroborated by the method validation parameters described in Sect. “Method validation parameters”, which include assessments of selectivity, accuracy, and matrix effect, all conducted in accordance with ICH M10 guidelines, showing that interference was not amongst the pharmaceuticals under study and the pharmaceutical excipients.

**Table 4:** Determination of dosage form by the developed HPTLC method and standard addition technique.

Pharmaceutical formulatio$\:{n}^{a}$ (Mean ± STD)	Meloxicam	104.58 ± 1.73
	Florfenicol	98.97 ± 0.11
Standard addition$\:{n}^{b}$ (Mean ± STD)	Meloxicam	103.92 ± 1.70
	Florfenicol	102.41 ± 0.56

(a) Average of six determinations (proposed concentrations were 4 µg/band for FLR and 0.05 µg/band for MEL).

(b) average of four determinations standard addition samples (the added concentrations to DF were 3.2, 4, and 4.8 µg/band for FLR, 0.04, 0.05, and 0.06 µg/band for MEL).

#### The removal recovery

By contrasting the peak area of the drugs extracted from the muscle that was spiked with that of pure standards, the extraction recovery was ascertained. Three distinct concentrations (QCs) were used to assess extraction recovery for each medication, and the results were displayed as a percentage recuperation±%RSD. In the case of the spiked muscle samples, the extraction recoveries ranged from 98.37 ± 0.07to 100.73 ± 0.05 for MEL, and from 98.86 ± 0.07 to 101.05 ± 0.30 for FLR. The results in Table 5 confirmed the efficiency and reproducibility of the extraction process.

**Table 5:** The extraction recovery results of meloxicam and florfenicol in spiked cattle muscles.

MEL		FLR	
Concentration(µg/band)	Recovery%±RSD%	Concentration	Recovery%±RSD%
0.30	100.34 ± 0.17	2.00	101.05 ± 0.30
1.50	98.37 ± 0.07	4.00	99.04 ± 0.05
2.40	100.73 ± 0.05	7.00	98.86 ± 0.07
Mean ± % RSD	99.81 ± 0.90		99.65 ± 0.14

Note: RSD, relative standard deviation.

#### Limit of detection and limit of quantification

LOD and LOQ are utilized to assess the analytical sensitivity of the procedure. The intercept of standard deviations and the calibration curve slope were used to compute them. The LOD (3.3 SD/slope) and LOQ (10 SD/slope) formulas are applied. For MEL, the LOD and LOQ for the standard samples were(0.03, 0.09) µg/band (15, 45) µg/kg, while for FLR, they were (0.14, 0.44) µg/band (70, 220) µg/kg. The LOD and LOQ for MEL in the spiked muscle samples were(0.06, 0.17) µg/band(30, 85) µg/kg, and (0.18, 0.56)µg/band(90,280)µg/kg. For FLR. The results presented that the recommended method provided the high sensitivity needed to quantify the components under study, even when the components were present in the tissues under test at quantities that matched their MRL. The results validated the method’s accuracy and suitability for quantifying the medicines under study in their current dose form.

#### Stability studies

The stability of (MEL) and (FLR) in the muscle matrix was evaluated under two conditions: bench-top stability at room temperature for 8 h, and freeze–thaw stability over three cycles of freezing at − 20 °C for 12 h followed by thawing at room temperature. Quality control (QC) samples at three concentration levels were analyzed and compared to freshly prepared samples. Statistical analysis using paired t-tests showed no significant differences in recovery between stored and fresh samples (*p* > 0.05). This indicates that the muscle matrix does not adversely affect the stability of MEL or FLR under the tested conditions. As summarized in Table 6, all recovery values were within ± 15% of nominal concentrations, complying with ICH M10 guideline acceptance criteria, thus confirming the analytes’ stability.

**Table 6:** Stability of MEL and FLR in muscle matrix under freeze–thaw and bench-top conditions using the proposed HPTLC method.

	Recovery$\:{\varvec{\%}}^{\varvec{a}}$		
	Concentration (µg/band)	Three freeze-thaw cycle$\:{s}^{b}$	Bench-top stability
MEL	0.30	100.03	98.63
	1.50	98. 06	97.17
	2.40	100.38	95.97
Mean ±%RSD		99.49 ± 0.11	97.26 ± 0.16
FLR	0.50	101.01	97.85
	2.00	95.96	94.24
	7.00	98.01	93.68
Mean ±%RSD		98.32 ± 0.16	95.26 ± 0.20

(a)average of three determinations.

(b)Freezing was done at −20◦C.

Note: Recovery values compared to freshly prepared QC samples using paired t-tests; differences were not statistically significant (p > 0.05).

#### Robustness

Table 2 offers the results of carefully examining the robustness of the supposed HPTLC method through a series of small, carefully considered changes to various criteria, including detected wavelength, mobile phase ratios, and saturation time. This demonstrated that the marginally examined circumstances had no discernible impact on the$\:{\:R}_{f}$ of distinct analytes, confirming the robustness of the procedures.

#### System suitability parameter

These variables were tested using every chromatographic technique to assess system performance before or during the dissection of our constituent parts. Among them were symmetry factors, resolution, selectivity, and capacity factor. The results appear in Table 7.

**Table 7:** Parameters of system suitability of the developed HPTLC method for the determination of meloxicam and florfenicol.

Parameter	Muscle	ESO	MEL	FLR	Reference value
Capacity factor (K^’^)	0.25	1.85	7.75	12.20	˃1.00
Tailing factor	-	1.10	1.07	1.00	≈ 1.00
Resolution (Rs)	-	-	6.21	4.45	≥ 1.50
Selectivity (α)	-	7.40	4.19	1.57	≥ 1.00

### Statistical comparison to the stated method

A statistical comparison was conducted between our proposed HPTLC method and previously reported TLC-densitometric methods for (MEL) and (FLR)^29–31^. We evaluated the recovery values using a Student’s t-test and an F-test at a 95% confidence level to assess differences in accuracy and precision. As shown in Table 8, the calculated t and F values for both drugs were below the critical limits ($\:{t}_{tab}$​ = 2.306; $\:{F}_{tab}$​ = 6.388), which confirms there are no statistically significant differences in either accuracy or precision between our method and the previous ones.

**Table 8:** Statistical analysis of the proposed HPTLC method and the reported methods for the determination of meloxicam and florfenicol in pure form.

	MEL		FLR	
Parameters	HPTLC method	Reported method	HPTLC method	Reported method
Mean	98.90	100.30	100.84	101.40
SD	0.89	1.51	1.35	2.08
Variance	0.79	2.28	1.82	4.32
N	5.00	5.00	5.00	5.00
Student’s t-test (2.306)	0.80	-	0.97	-
F-test (6.388)	2.91	-	1.51	-

Beyond its comparable performance, our HPTLC method offers several key advantages. It uses greener solvents and is reliable in a complex biological matrix like muscle tissue. In contrast, the previously reported methods were only validated for analyzing pharmaceutical tablets. These practical benefits make our method a more suitable option for routine monitoring of veterinary drug residues in food safety laboratories.

### Greenness and blueness evaluation of the supposed HPTLC method

#### Greenness evaluation

##### Analytical greenness metric approach

**Fig. 3:**
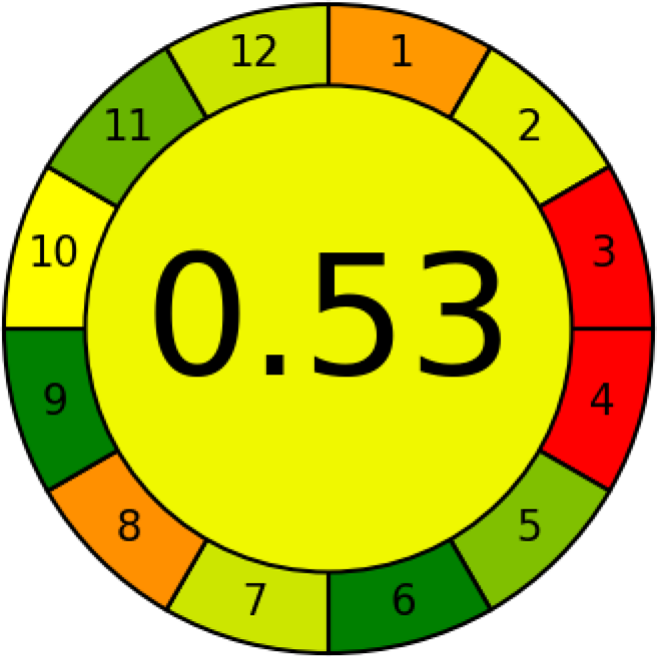
Analytical greenness metric approach (AGREE)-colored pictogram of the developed HPTLC method.

The AGREE^32^ is one instrument for estimating how green the recommended strategies are. This ranges from zero to one, with the overall score being a fraction of one. Its fundamental ideas address several aspects Like technique simplicity, waste generation, and energy usage. The whole score for the AGREE system was 0.53, as shown in Fig. 3.

##### Analytical greenness metric for sample Preparation

**Fig. 4:**
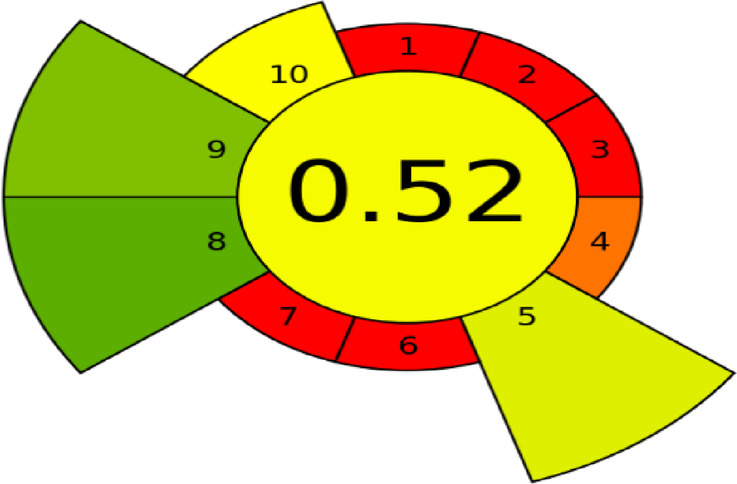
Analytical greenness metric for sample preparation (AGREE prep) colored pictogram of the developed HPTLC method.

The AGREE prep tool assesses sample preparation methods against ten key principles, each scored from 0 (least sustainable) to 1 (most sustainable). These principles include sample size, the number of preparation steps, the hazards associated with solvents, energy consumption, and operator safety. The AGREE prep system received an overall score of 0.52, as depicted in Fig. 4.

##### Green analytical procedure index

**Fig. 5:**
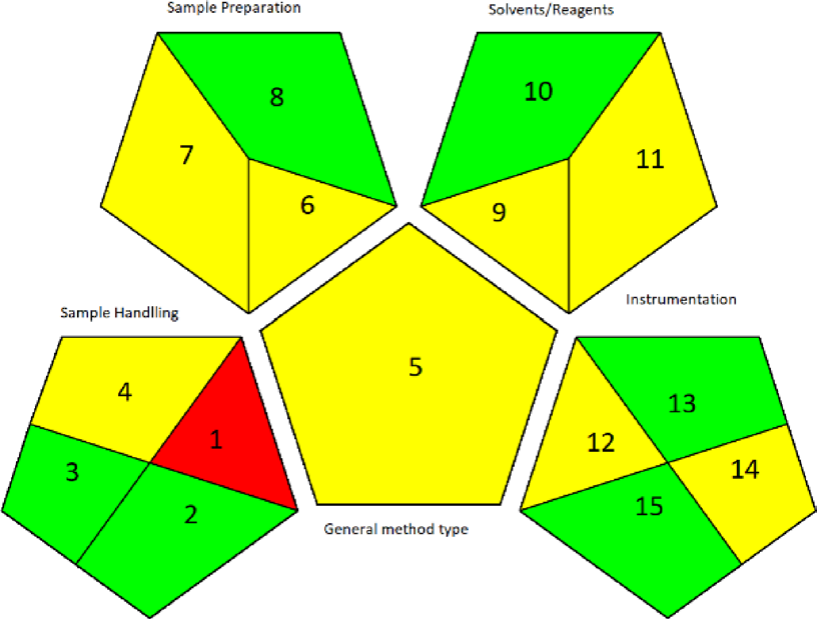
Green analytical procedure index (GAPI)-colored pictogram of the developed HPTLC method.

GABI^33^ is a trustworthy tool for evaluating all aspects of the analytical process, from sample preparation to analysis completion. The GAPI system is divided into five main parts, each of which describes a different set of parameters. Gathering, preserving, transporting, and storing samples come first. Sample preparation is under the second category. The third is about reagents and solvents. Instrumentation comes in fourth, followed by the general method type in fifth. Depending on the safety process in each part, the GAPI approach is displayed as a three-colored pictogram (Fig. 5).

#### Blueness evaluation

##### Blue applicability grade index

**Fig. 6:**
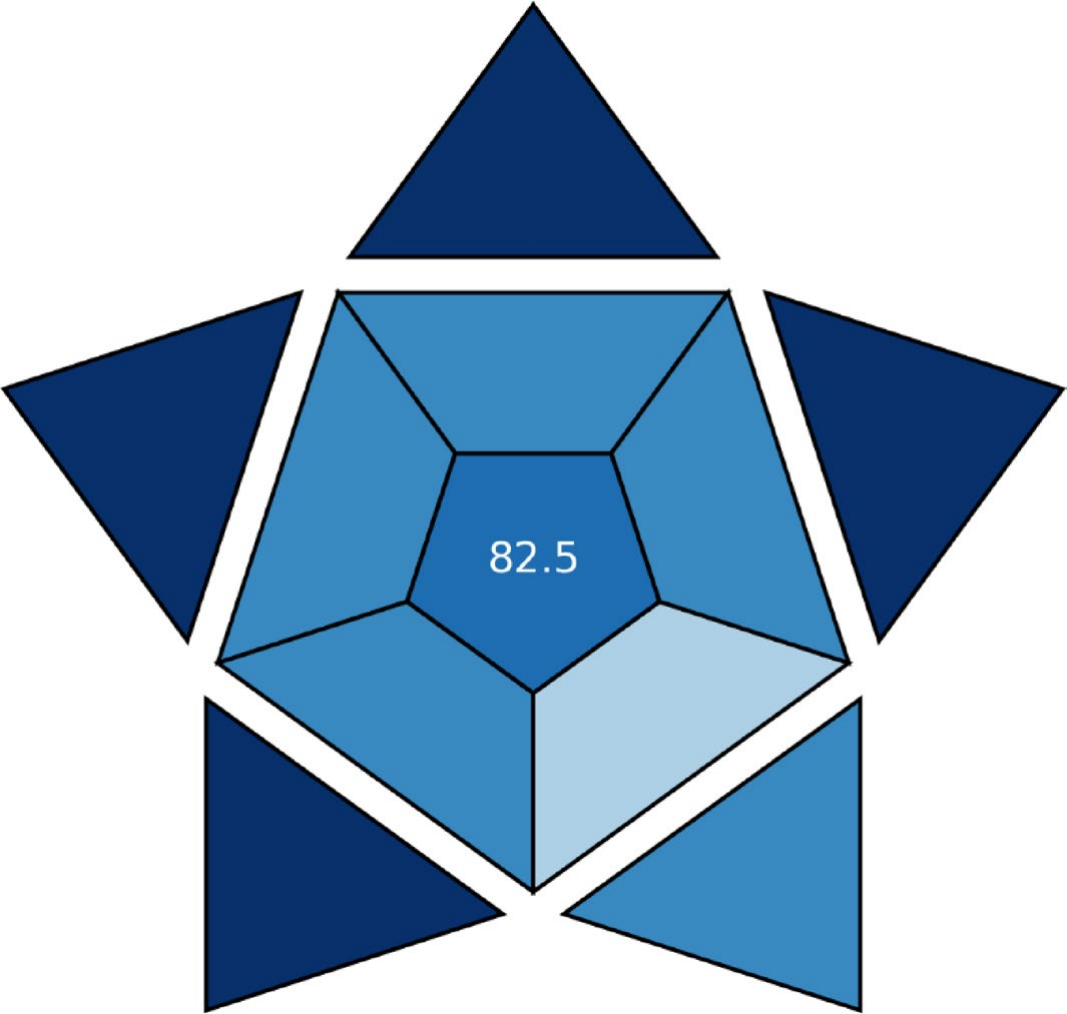
Blue Applicability Grade Index (BAGI)-colored pictogram of the developed HPTLC method.

A novel statistic for evaluating an analytical technique’s applicability in real-world situations is the BAGI^34^. This index assesses ten important factors. After evaluating these factors, an asteroid diagram is produced as a visual depiction with the associated score. This pictogram’s color spectrum shows how closely the approach adheres to predetermined standards. In particular, white denotes no alignment, Light blue denotes low alignment, blue denotes moderate alignment, and dark blue denotes great alignment. A total score for the analytical procedure is indicated by the number in the middle of the BAGI pictogram. The spectrum ranges from 25 to 100. Excellent method performance is indicated by a score of 100, whilst low applicability is indicated by a score of 25. Using the BAGI tools, the suggested HPTLC method was evaluated. Figure 6 shows that it received a favorable score of 82.5, indicating strong applicability.

#### Whiteness evaluation

**Fig. 7:**
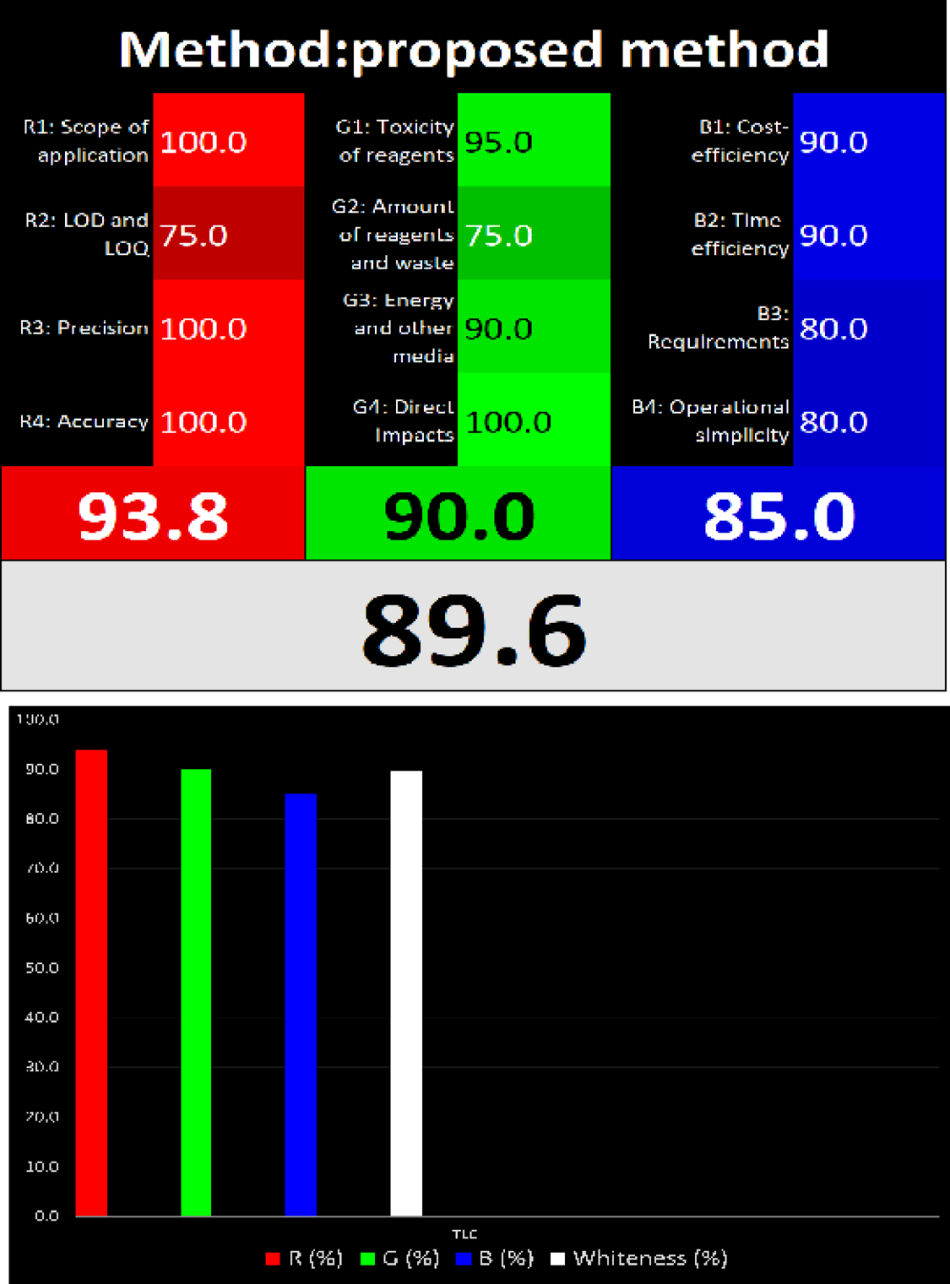
RGB12 algorithm for the suggested HPTLC method.

The proposed HPTLC method demonstrated excellent performance, achieving scores of 93.8%, 90.0%, and 85.0% for the red, green, and blue color metrics, respectively. These high scores underscore the method’s superior sensitivity and its efficient use of solvent, time, and energy. Furthermore, the HPTLC method attained an overall whiteness score of 89.6%, affirming its appropriate quality, strong greenness, and practical applicability, as depicted in Fig. 7.

#### Contextualizing greenness, whiteness, and blueness: A Literature-Based perspective

The developed HPTLC method achieved an AGREE score of 0.53, a BAGI score of 82.5, and an RGB12 score of 89.6, demonstrating a well-balanced profile of greenness, analytical quality **(**whiteness), and practical feasibility (blueness). Importantly, the linearity ranges for FLR and MEL are more sensitive than those reported for RP-HPLC and HPLC methods. As shown in Table 9, this study uniquely provides an assessment of blueness and whiteness, which were not evaluated in the previously published methods, highlighting the comprehensive sustainability evaluation of the developed method.

**Table 9:** Comparative evaluation of the developed HPTLC method with reported RP-HPLC-DAD and HPLC methods.

Parameter	Developed HPTLC	Reported RP-HPLC-DAD^35^	Reported HPLC^36^
Linearity range	FLR (0.50-9.00) (µg/band) MEL (0.03–3.00) (µg/band)	FLR (0.05–20.00) (µg/mL)	MEL (5.00–100.0) µg/mL
Mobile phase	glacial acetic acid, methanol, tri-ethyl amine, and ethyl acetate (0.05: 1.00: 0.10: 9.00, by volume)	component A (water and acetonitrile) 80: 20 v/v; pH = 3.50 with phosphoric acid) and component B (acetonitrile)	acetonitrile: 0.05 M potassium dihydrogen phosphate buffer at pH 5.00
Matrix	Cattle muscle	Pig urine	Tablets
Cost	Low: cheap solvent and plate	High: expensive solvent, extraction, and column	High: expensive solvent and column
AGREE pictogram			
GABI pictogram			

## Conclusion

With ESO serving as an IS, the HPTLC method was developed to assess MEL and FLR concurrently in binary mixtures and spiked muscle samples, taking into consideration the maximum residue limits of these drugs in cattle meat according to the European Commission and exceeding these limits cause bacterial resistance and liver problem to human The method was incredibly sensitive, quick, inexpensive, easy to use, and environmentally benign. It also caused less harm to the environment. All validation parameters satisfied the ICH acceptance criteria for the validation of bioanalytical methods. Moreover, it has been shown that the proposed method for estimating the combined MEL and FLR in the Zeleris^®^ injection solution is precise and effective. The recommended strategy was the first HPTLC densitometric method ever developed, with a straightforward preparation procedure and excellent sensitivity for assessing the combination alternative to more costly chromatographic methods.

## Data Availability

The datasets used and/or analysed during the current study are available from the corresponding author on reasonable request.
